# Sex-specific nociceptor modulation of the apical periodontitis transcriptome

**DOI:** 10.3389/fmolb.2024.1338511

**Published:** 2024-02-09

**Authors:** Katherine V. Lillis, Ruta Grinceviciute, Anibal Diogenes

**Affiliations:** Department of Endodontics, University of Texas Health at San Antonio, San Antonio, TX, United States

**Keywords:** transcriptomics, bone, apical periodontitis, dental infection, nociceptors

## Abstract

**Introduction:** Apical periodontitis (AP) is a painful disease that develops quickly following dental infections and is primarily characterized by robust inflammation surrounding the tissues of the affected tooth, resulting in disruption of bone homeostasis and periradicular bone loss. Moreover, there are distinct clinical presentations, symptoms, and responses to AP treatment between male and female subjects, creating a desperate need to further understand the sex-specific mechanisms of AP.

**Methods:** With the growing evidence that nociceptors modulate AP development, we utilized RNA sequencing in nociceptor-ablated (Nav1.8 ^cre+/−^, diphtheria toxin A^lox+/−^) transgenic mice to study the nociceptor regulation of the periapical lesion transcriptome using a rodent model of AP in female mice over 14 days.

**Results:** Overall, we found that female mice exhibit unique patterns of differentially expressed genes throughout AP infection compared to male mice and that the expression of these genes is regulated by nociceptors. Additionally, nociceptor ablation results in a more significant enrichment of biological processes related to immune responses earlier compared to cre-control (Nav1.8 ^cre+/−^) females and greater expression of genes involved in inflammatory processes and osteolytic activity.

**Discussion:** Therefore, while nociceptor ablation augments inflammatory and bone resorption responses in both males and females in a mouse model of AP, transcriptomic analyses demonstrate that the mechanisms through which nociceptors modulate AP are distinct between sexes. These studies will provide the foundation needed to study further mechanisms of sex differences in AP, an area with a desperate need for investigation to treat current AP patients. Understanding these mechanisms can ultimately inform treatment options to alleviate suffering for millions of patients suffering from AP.

## 1 Introduction

Apical periodontitis (AP) is a painful disease that develops quickly following dental infections and is primarily characterized by robust inflammation surrounding the affected tooth, disrupting bone homeostasis, thereby resulting in bone loss (Graunaite et al., 2012). Moreover, there are distinct clinical presentations, symptoms, and responses to AP treatment between male and female individuals (Polycarpou et al., 2005; Khan et al., 2007; Estrela et al., 2011). Female subjects have been shown to have greater AP prevalence than male subjects, as determined by the frequency of apical radiolucency (Berlinck et al., 2015). Clinical studies have reported a consistent sex difference in odontalgia, with females reporting greater pain (Polycarpou et al., 2005; Estrela et al., 2011), greater analgesic consumption (Nusstein and Beck, 2003), and less analgesic pain relief compared to males (Nusstein and Beck, 2003). Overall, clinical reports are consistent with those in preclinical studies, in which mechanical allodynia was assessed in mice, following a pulp exposure procedure, where female mice exhibited significantly lower baseline mechanical thresholds compared to male mice (Mohaved et al., 2020). These clinical and preclinical studies demonstrate a desperate need to further understand the sex-specific mechanisms of AP to enhance future treatment options.

In recent years, preclinical studies have focused on investigating key hallmarks of AP: periapical inflammation and bone loss. As such, there has been extensive work studying the crosstalk between immune and bone cells throughout the development of the disease (Silva et al., 2007; Garlet, 2010; Benedetto et al., 2013; Yucel-Lindberg and Båge, 2013). Dental infections leading to AP trigger the recruitment of immune cells and their subsequent release of inflammatory mediators, such as cytokines, chemokines, prostaglandins, and proteolytic enzymes (Benedetto et al., 2013; Yucel-Lindberg and Båge, 2013). This inflammatory environment stimulates bone resorption via differentiation and activation of osteoclasts, primarily resulting from increased levels of the receptor activator of NF-κB (RANKL), the cytokine responsible for initiating osteoclastogenesis (Benedetto et al., 2013).

Our laboratory has shown that the regulation of AP inflammation and osteolytic activity is tightly regulated by nociceptors densely innervating the teeth and surrounding periapical tissue, demonstrating their active role in the disease (Austah et al., 2022; Lillis et al., 2023). First, we established that nociceptor ablation increases periapical bone loss and the early influx of immune cells *in vivo* using a mouse model of AP (Austah et al., 2022). We also demonstrated that nociceptors enhance osteoblast mineralization and inhibit osteoclast resorption activity *in vitro* (Austah et al., 2022). Later, we showed that nociceptor ablation enhances the expression of inflammatory genes throughout the course of infection using transcriptomic analyses in male mice (Lillis et al., 2023). Given the unique clinical and preclinical presentations of AP in male and female subjects, we used RNA sequencing of periapical lesions in a mouse model of the disease to test the hypothesis that nociceptors modulate the periapical transcriptome in a sex-specific manner to further study sex-specific changes in the transcriptomic profile of AP tissue and its regulation by nociceptors in the present study.

## 2 Materials and methods

We generated nociceptor-ablated transgenic mice, as described previously, following the UT Health San Antonio IACUC and ARRIVE guidelines (Austah et al., 2022; Lillis et al., 2023). Mice were housed two to five per cage with food and water available *ad libitum*. For all experiments, we used 8–12-week-old female mice, and identical experiments were carried out simultaneously in a male cohort, published earlier (Lillis et al., 2023). In brief, cre-control (Nav1.8 ^cre+/−^) and nociceptor-ablated (Nav1.8 ^cre+/−^ DTA^lox+/−^) mice were generated by crossing heterozygous Nav1.8^cre+^ mice with homozygous Rosa26^tm1(DTA)Lky+^, as previously established (Stirling et al., 2005; Austah et al., 2022; Lillis et al., 2023).

We used a well-characterized model of apical periodontitis in mice, as described previously (Austah et al., 2022; Lillis et al., 2023). In brief, we performed a pulp exposure in the mandibular and maxillary first molars and allowed for infections to develop over the course of 0, 7, or 14 days (*n* = 3-4 mice/strain/timepoint, *n* = 20 mice total). Then, at the respective time points, mice were briefly anesthetized with isoflurane and euthanized via cervical dislocation. The first molars were dissected and immediately snap-frozen in liquid nitrogen. As previously described, we extracted RNA using the RNeasy Kit (QIAGEN; Valencia, CA), according to the manufacturer’s recommendation (Lillis et al., 2023). RNA was quantified using the NanoDrop instrument (Thermo Scientific; Rockford, IL) and stored at −80°C.

Total RNA was submitted to the Genome Sequencing Facility at UT Health San Antonio, where RNA sequencing and blinded bioinformatics analysis were performed (Mecklenburg et al., 2020). Total RNA was sequenced and analyzed, as described previously (Lillis et al., 2023). In brief, the KAPA Stranded RNA-Seq Kit with RiboErase (HMR) (Cat. #, KR1151, KAPA Biosystems) was used to prepare the RNA-Seq library. The TopHat2 default settings were used to align RNA-seq FastQ reads with the UCSC mouse build mm9 reference genome, and the aligned BAM files were sorted (SAMTools) and then processed using HTSeq-count to obtain the counts per gene to generate reads per kilobase of the transcript per million mapped reads (RPKM). We then used the R package “DESeq” to normalize data and perform pairwise comparisons within strains between days 0, 7, and 14 and between strains at each time point. The cutoffs for up- and downregulated differentially expressed genes (DEGs) were fold change (FC) > 1.5 with a *p*-value <0.05. Gene Ontology biological process analyses were performed, as described previously (Austah et al., 2022; Lillis et al., 2023). We used the PANTHER overrepresentation test (Mi et al., 2019) to analyze enriched biological processes for DEGs between time points, including generation of the fold enrichment score, number of genes per process, and *p*-values (Bonnot et al., 2019).

### 2.1 Real-time PCR

We validated our findings with real-time PCR, as described previously (Lillis et al., 2023). In brief, cDNA was synthesized from total RNA using the High-Capacity RNA-to-cDNA kit, based on the manufacturer’s instructions (Thermo Fisher Scientific; Waltham, MA). We then used the TaqMan™ Fast Advanced Master Mix (Thermo Fisher Scientific; Waltham, MA) and TaqMan™ gene expression assays to conduct real-time PCR experiments on an ABI 7500 Fast Real-Time PCR System (Thermo Fisher Scientific; Waltham, MA). Relative gene expression was determined using the comparative delta–delta cycle threshold method (ΔΔCt), normalizing each gene of interest to GAPDH and cre-control Day 0 samples as the calibrator, as previously described (Schmittgen and Livak, 2008). Statistical analysis was performed using unpaired t-tests using GraphPad Prism software with the statistical significance set at *p* < 0.05.

## 3 Results

### 3.1 Nociceptor-ablated females express a greater number of differentially expressed genes after 14 days of infection compared to cre-control females

**FIGURE 1:**
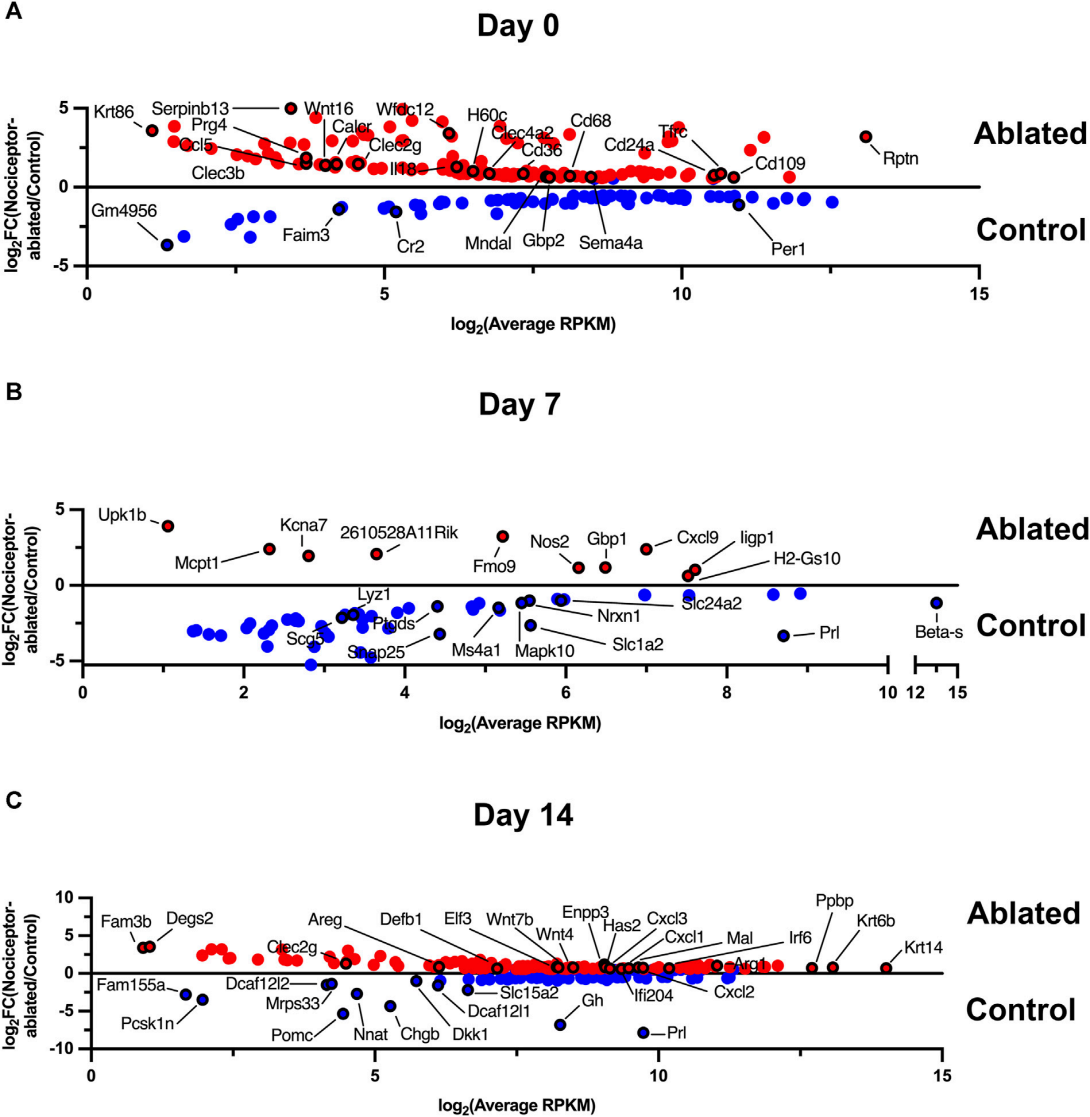
Nociceptor-ablated females express a greater number of differentially expressed genes after 14 days of infection compared to cre-control females. Enriched gene expression in either cre-control (Control) or nociceptor-ablated (ablated) females at day 0 **(A)**, day 7 **(B)**, and day 14 **(C)**. Differentially expressed (genes) are plotted as expression in average reads per kilobase of transcript per million mapped reads (RPKM) against the fold change (FC) between nociceptor-ablated expression and cre-control expression. DEGs were defined as fold change (FC) > 1.5 and *p* < 0.05. *n* = 3–4/strain/time point.

Immediately after pulp exposure, there were 148 differentially expressed genes (DEGs; fold change [FC] >1.5 and *p* < 0.05) in nociceptor-ablated females compared to cre-control females (28 DEGs) at day 0 (Figure 1A). After 7 days of infection, nociceptor-ablated females exhibited 12 DEGs in contrast to the 51 DEGs in the cre-control periapical lesions (Figure 1B). Cre-control females had greater expression of genes, including prolactin (*Prl*). However, at 14 days, nociceptor-ablated females showed a more significant number of DEGs at 128 genes total, yet cre-control females exhibited 16 DEGs at this time point (Figure 1C). Genes with greater expression in nociceptor-ablated females after 14 days included the wingless-type MMTV integration site family (Wnt), member 7B (*Wnt7b*), *Wnt4*, chemokine (C-X-C motif) ligand 2 (*Cxcl2*), and *Cxcl1*. Interestingly, cre-control females demonstrated greater *Prl*, growth hormone (*Gh*), and dickkopf Wnt signaling pathway inhibitor 1 (*Dkk1*) levels within apical periodontitis lesions at 14 days post-pulp exposure.

### 3.2 Male and female differentially expressed genes share a minimal overlap except for nociceptor-ablated mice after 14 days of infection

**FIGURE 2:**
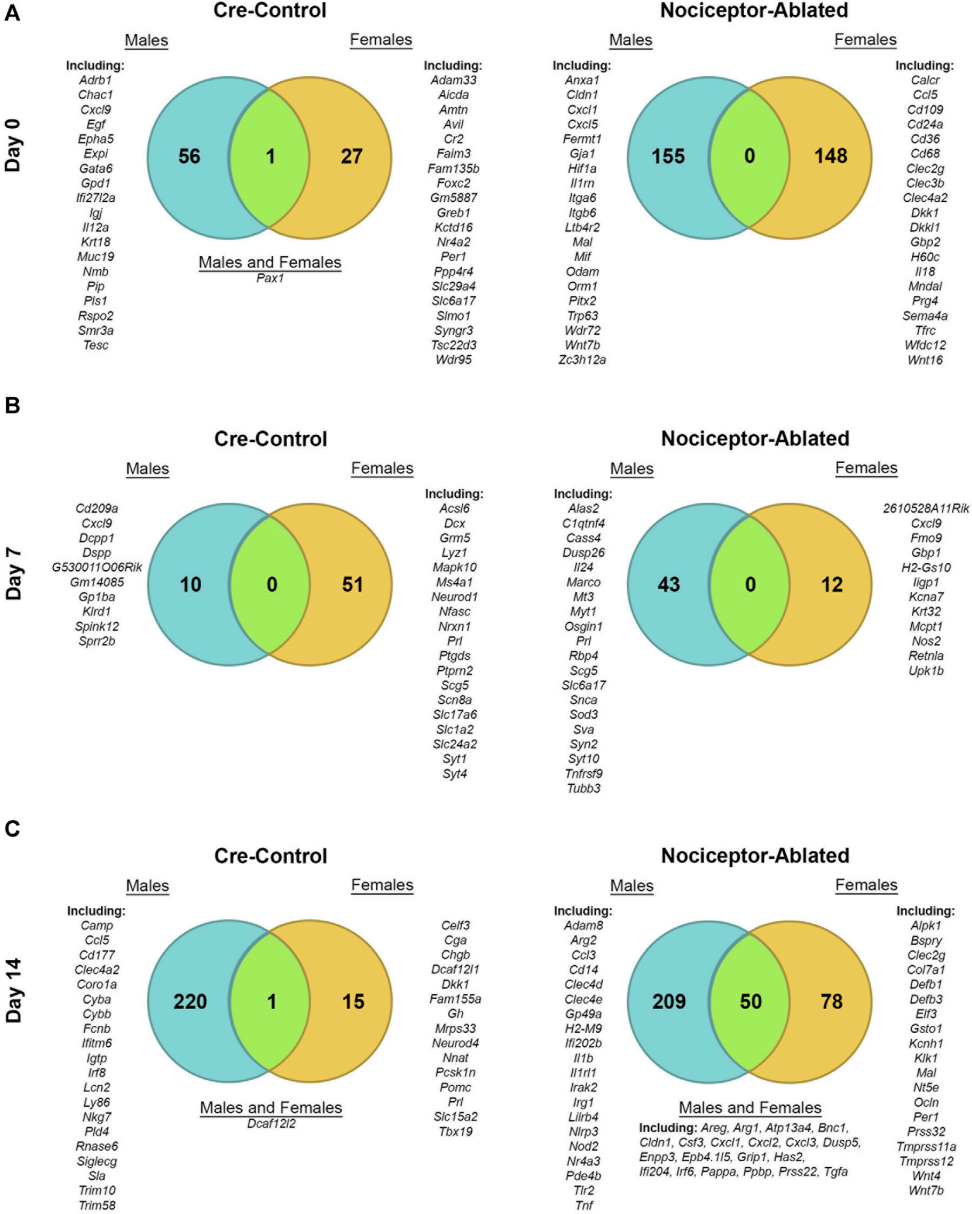
Male and female differentially expressed genes share a minimal overlap except for nociceptor-ablated mice after 14 days of infection. Venn diagrams depicting the total number of DEGs enriched in either cre-control (left column) or nociceptor-ablated (right column) mice for males and females at day 0 **(A)**, day 7 **(B)**, and day 14 **(C)**. DEGs were defined as fold change (FC) > 1.5 and *p* < 0.05. Examples of male DEGs are listed on the left; examples of female DEGs are listed on the right; and common DEGs within each strain are listed in the middle (if applicable). A full male transcriptomic dataset can be found at GEO #GSE205195 (Lillis et al., 2023). *n* = 3–4/sex/strain/time point.

We have previously reported male AP lesion transcriptomics at 0-, 7-, and 14-day post dental infection induction in nociceptor-ablated and cre-control mice (Lillis et al., 2023). When compared to males, females exhibited distinct patterns of DEGs at each time point, and nociceptors modulated these effects. At day 0, female cre-control mice had 27 DEGs, whereas male cre-control mice expressed 56 DEGs, with only paired box 1 (*Pax1*) shared between sexes (Figure 2A, left). For DEGs from nociceptor-ablated mice at day 0, females had 148 DEGs, while males had 155 unique DEGs, with no overlap between sexes (Figure 2A, right). After 7 days of infection, female cre-control mice exhibited 51 DEGs (e.g., *Prl*) and males had 10 DEGs (e.g., dentin sialophosphoprotein [*Dspp*]), with no DEGs in common (Figure 2B, left). In contrast, nociceptor-ablated females presented fewer DEGs at day 7, with only 12 DEGs, in contrast to 43 DEGs from nociceptor-ablated males (Figure 2B, right). At day 14 post-pulp exposure, cre-control females showed fewer DEGs, with 15 DEGs (e.g., *Prl* and *Gh*), compared to the 220 DEGs male cre-control mice possessed (Figure 2C, left). However, cre-control males and females shared one DEG in common, DDB1 and CUL4-associated factor 12-like 2 (*Dcaf12l2*). For nociceptor-ablated mice, day 14 was the time point where the most DEGs were shared between sexes, with 50 DEGs in common between males and females (Figure 2C, right). Overall, nociceptor-ablated males had 209 DEGs at day 14 (e.g., interleukin 1 beta [*Il1b*] and tumor necrosis factor [*Tnf*]), while females had 78 DEGs (e.g., *Wnt4* and *Wnt7b*).

### 3.3 Nociceptor-ablated females exhibit unique changes in gene expression over the course of AP infection compared to cre-control females

**FIGURE 3:**
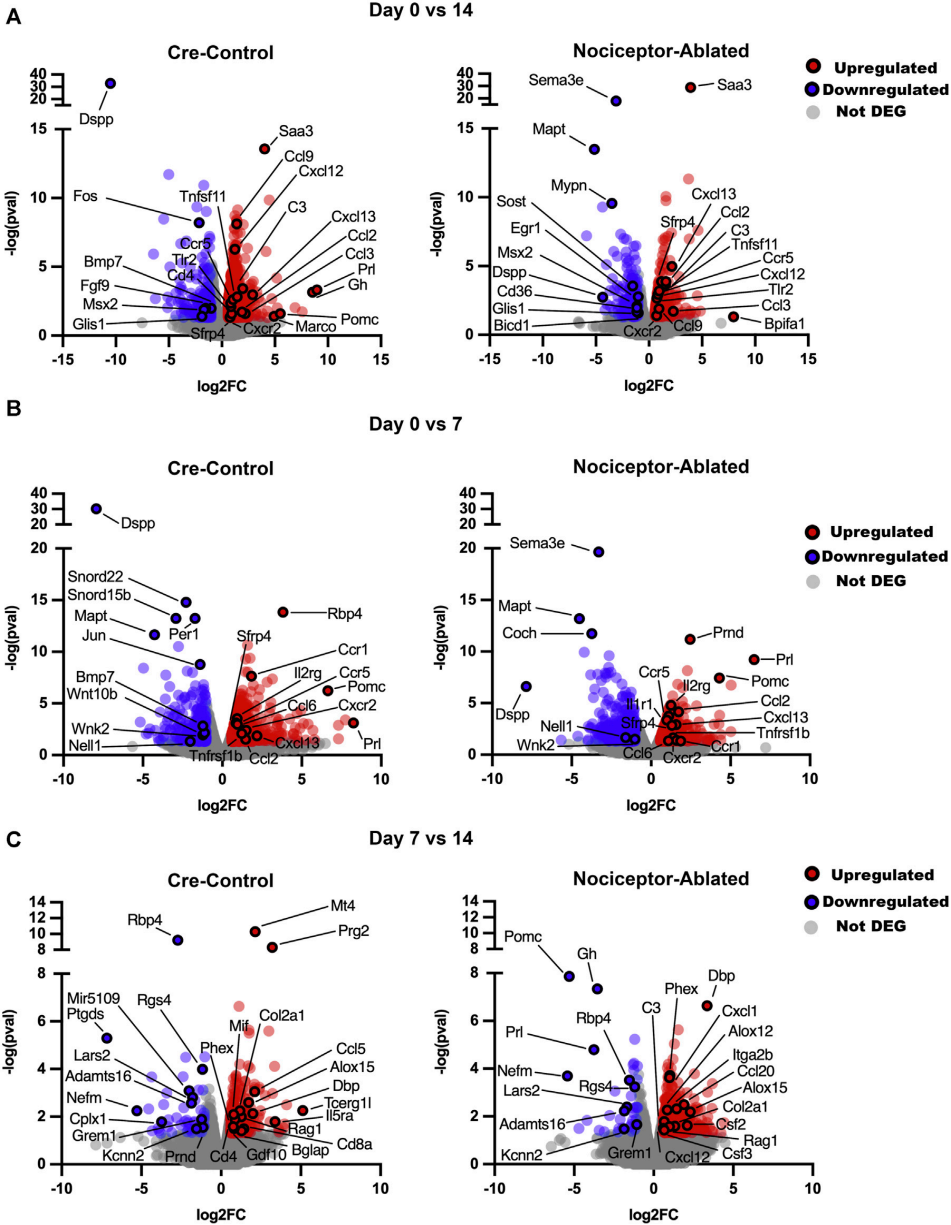
Nociceptor-ablated females exhibit unique changes in gene expression over the course of AP infection compared to cre-control females. Volcano plots comparing gene expression over time for cre-control and nociceptor-ablated female mice. Data are presented as FC (later vs earlier time point) against the *p*-value for day 0 vs. 14 **(A)**, day 0 vs. 7 **(B)**, and day 7 vs. 14 **(C)**. DEGs were defined as FC > 1.5 and *p* < 0.05. *n* = 3–4/strain/time point.

After 14 days of infection, nociceptor-ablated females exhibited 158 unique upregulated (FC > 1.5, *p* < 0.05) genes, whereas cre-control females had 614 upregulated genes when compared to day 0 expression (Figure 3A). A total of 170 genes were upregulated in both nociceptor-ablated and cre-control females between 0 and 14 days. Among these common upregulated genes, nociceptor-ablated mice had greater FC for secreted frizzled-related protein 4 (*Sfrp4*), tumor necrosis factor (ligand) superfamily, member 11 (*Tnfsf11*), and chemokine (C-C motif) ligand 3 (*Ccl3*). Notably, *Prl* and *Gh* were upregulated only in AP of female mice between 0 and 14 days. During this timeframe, 115 genes were downregulated uniquely in nociceptor-ablated females, and 184 were downregulated only in cre-control females, while 71 genes were commonly downregulated in both strains. Of the common downregulated genes, cre-control females had greater FC downregulation for genes, including *Dspp* and fibroblast growth factor 9 (*Fgf9*). Nociceptor-ablated females showed exclusive downregulation of genes like sclerostin (*Sost*), whereas genes like bone morphogenetic protein 7 (*Bmp7*) were only downregulated in cre-control.

Upon investigating changes in the gene expression earlier in AP infection, nociceptor-ablated and cre-control females had upregulation of 297 common genes between 0 and 7 days (Figure 3B). Nociceptor-ablated females presented exclusive upregulation of 240 genes, whereas cre-control females had upregulation of 478 unique genes. Of the commonly upregulated genes, nociceptor ablation led to greater FC for genes including *Sfrp4* and *Ccl2*. Cre-control females had greater FC for upregulated genes including *Cxcl13* and *Prl*. Genes exclusively upregulated in nociceptor-ablated females included *Tnf*, *Ccl3*, and *Il1b*, and cre-control females showed upregulation of genes including *Tnfsf11*, the tumor necrosis factor receptor superfamily, member 11a, NFKB activator (*Tnfrsf11a*), matrix metallopeptidase 8 (*Mmp9*), cathepsin K (*Ctsk*), acid phosphatase 5, tartrate-resistant acid phosphatase 5 (*Acp5*), and *Il6*. For genes downregulated between 0 and 7 days, there were 135 genes shared between the two strains, and nociceptor-ablated females had 270 unique downregulated genes compared to 233 for cre-control, where *Dspp* was more downregulated in nociceptor-ablated females. Notably, only cre-control females had downregulation of *Wnt10b* and *Bmp7* between 0 and 7 days.

Later in the AP infection, 51 genes were commonly upregulated between nociceptor-ablated and cre-control females when comparing expression between 7 and 14 days (Figure 3C). Nociceptor-ablated females expressed 279 exclusively upregulated genes, and cre-control females had a unique upregulation of 183 genes. Of the shared genes, the phosphate-regulating endopeptidase homolog, X-linked (*Phex*) and collagen, type II, alpha 1 (*Col2a1*) showed a greater fold change increase in nociceptor-ablated females compared to cre-control. Only nociceptor-ablated females demonstrated a downregulation of *Prl*, *Gh*, and pro-opiomelanocortin-alpha (*Pomc*) between 7 and 14 days.

### 3.4 Nociceptor ablation results in a greater enrichment of biological processes related to immune responses compared to control females

**FIGURE 4:**
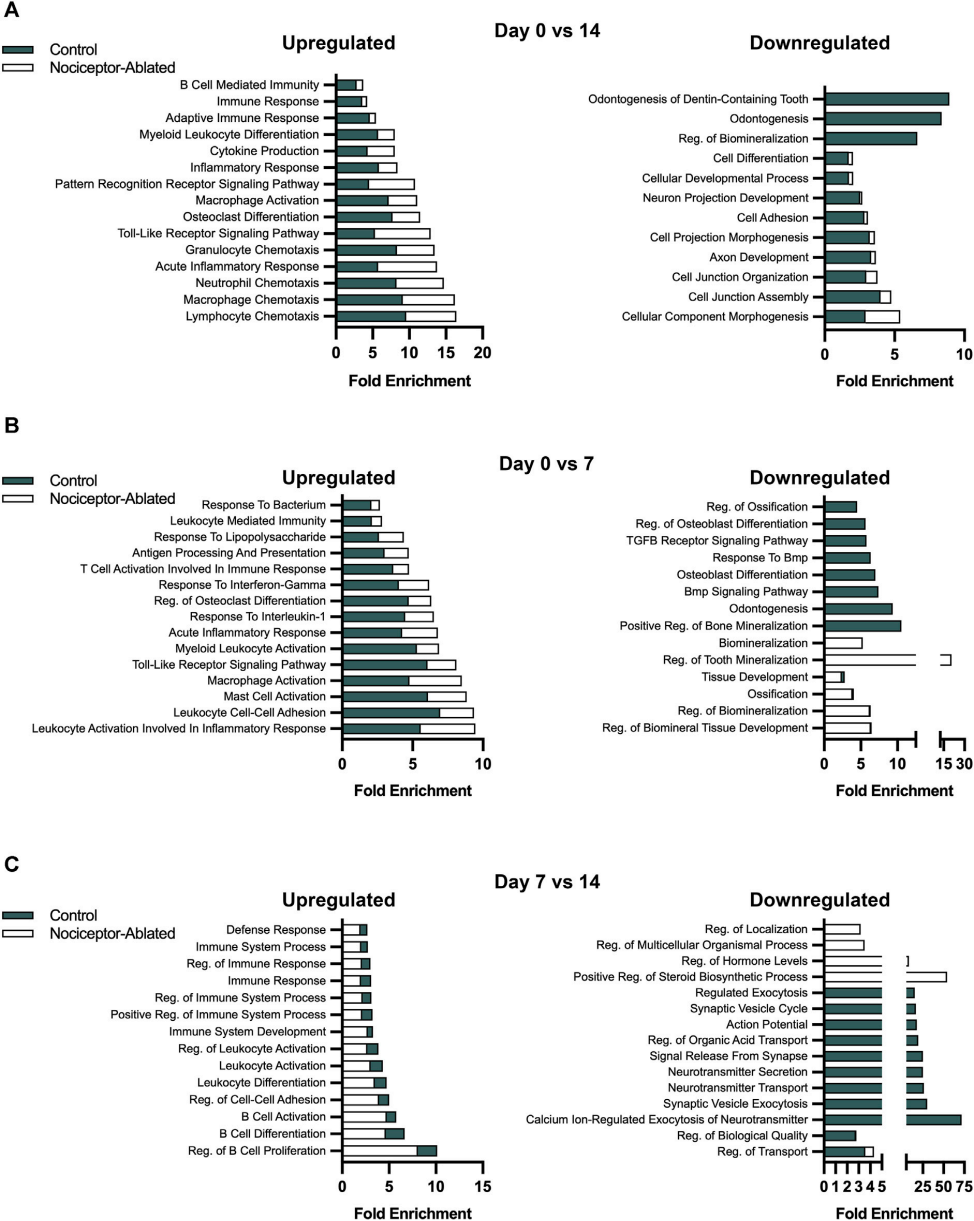
Over the course of AP infection, nociceptor ablation results in a greater enrichment of biological processes related to immune responses earlier compared to cre-control females. Selected biological processes for cre-control and nociceptor-ablated upregulated (left) and downregulated (right) DEGs. The fold enrichment score is plotted for each selected biological process for day 0 vs. 14 **(A)**, day 0 vs. 7 **(B)**, and day 7 vs. 14 **(C)**. N = 3–4/strain/time point.

After 14 days of infection, Gene Ontology biological processes (BPs) were enriched in both nociceptor-ablated and cre-control females. Between 0 and 14 days, nociceptor-ablated mice had a greater enrichment for processes related to the chemotaxis of lymphocytes, macrophages, neutrophils, and granulocytes (Figure 4A, left). Nociceptor-ablated mice also had ∼2.4× greater enrichment of processes, including the toll-like receptor signaling pathway, acute inflammatory response, and pattern recognition receptor signaling pathway. Additionally, nociceptor-ablated females had ∼1.5× more significant enrichment of osteoclast differentiation. For downregulated DEGs, only cre-control females had a substantial enrichment of odontogenesis, odontogenesis of the dentin-containing tooth, and regulation of biomineralization between 0 and 14 days (Figure 4A, right).

Earlier in the infection, nociceptor-ablated mice had enriched upregulation of biological processes related to immune responses and inflammation between 0 and 7 days following pulp exposure. Notably, many processes related to immune cell activation had greater upregulation enrichment in nociceptor-ablated females compared to cre-control (Figure 4B, left). For instance, mast cell activation, macrophage activation, and myeloid leukocyte activation were processes with a greater enrichment in nociceptor-ablated females. There was also ∼1.7× greater enrichment of leukocyte activation involved in inflammatory response and ∼1.3× greater enrichment of regulation of osteoclast differentiation in nociceptor-ablated mice. In contrast, many of the earlier downregulated biological processes in both strains involved mineralization (Figure 4B, right). Of the processes commonly downregulated, regulation of biomineral tissue development, regulation of biomineralization, ossification, and tissue development had a similar fold enrichment between nociceptor-ablated mice and cre-control.

During the later stages of the infection, cre-control mice started to exhibit greater expression of immune response biological processes compared to nociceptor-ablated mice, particularly for upregulated genes (Figure 4C, left). Between days 7 and 14, cre-control mice had a greater enrichment in processes related to B cells, including regulation of B-cell proliferation, differentiation, and activation. There was also a greater enrichment of leukocyte differentiation and activation. Furthermore, cre-control mice had enriched processes related to immune system process and immune response later in the infection compared to nociceptor-ablated mice. Conversely, the downregulated processes at this stage were more related to secretion and cellular communication pathways (Figure 4C, right).

Nociceptors regulated the expression of genes related to inflammation and bone homeostasis throughout the course of AP infection.

**FIGURE 5:**
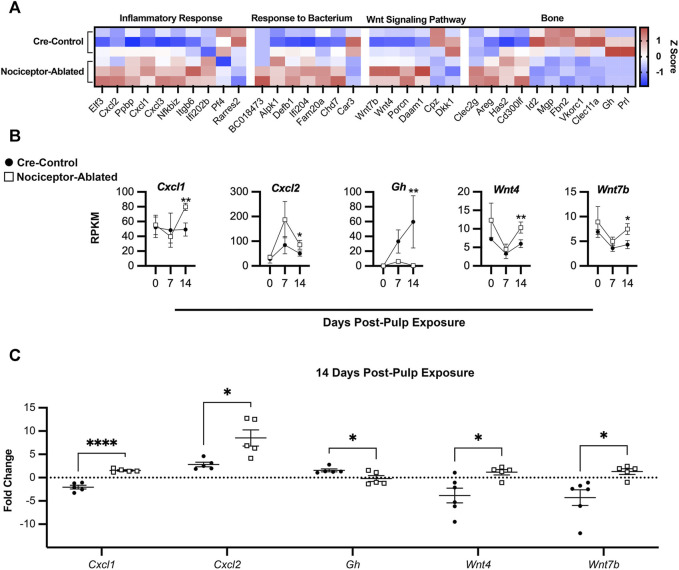
Nociceptors regulate the expression of genes related to inflammation and bone homeostasis throughout the course of AP infection. Heatmap of selected DEGs at day 14 based on the calculated Z-score of individual gene RPKM values separated by biological process for cre-control (top) and nociceptor-ablated (bottom) samples (n = 3/strain) **(A)**. Time course of gene expression throughout AP progression plotted as days post-pulp exposure vs. RPKM for selected genes for cre-control (circle) and nociceptor-ablated (square) mice **(B)**. RT-PCR validation of selected genes for cre-control (circle) and nociceptor-ablated (square) mice **(C)**. *n* = 3–4/strain/time point. *p < 0.05, **p < 0.01, and ***p < 0.001.

After 14 days of infection, there were prominent differences in the gene expression between nociceptor-ablated and cre-control females (Figure 5A). Within genes related to the inflammatory response, nociceptor-ablated mice had significantly greater expression of genes including *Cxcl1*, *Cxcl2*, and *Cxcl3*. Nociceptor-ablated mice also had greater expression of genes involved in response to the bacterium, while cre-control mice had greater expression of *Car3*. There were also significant differences within genes involved in bone metabolism. Within the Wnt signaling pathway, nociceptor-ablated mice had higher expression of *Wnt7b*, *Wnt4*, and porcupine O-acyltransferase (*Porcn*) after 14 days, whereas cre-control mice had greater expression of carboxypeptidase Z (*Cpz*) and Dickkopf Wnt signaling pathway inhibitor 1 (*Dkk1*). Further analyses of bone metabolism genes revealed that nociceptor-ablated mice had greater expression of C-type lectin domain family 2, member g (*Clec2g*), whereas cre-control females had significantly greater expression of *Prl* and *Gh* after 14 days (Figure 5B). Day 14 RT-PCR validation revealed similar patterns of gene expression, where nociceptor-ablated mice showed greater expression of *Cxcl1*, *Cxcl2*, *Wnt4*, and *Wnt7b*, whereas cre-control showed greater expression of *Gh* (Figure 5C).

## 4 Discussion

Apical periodontitis is a prevalent, debilitating disease affecting the lives of millions of patients every day. Despite a greater prevalence and unique presentation in females, there has been a gap in knowledge regarding the mechanisms driving sex-specific differences in the disease (Polycarpou et al., 2005; Berlinck et al., 2015). In this study, we utilized RNA sequencing to study the transcriptomic profile of periapical lesions in female mice over the course of 14 days. Furthermore, we investigated the role of nociceptors in modulating the disease in a sex-specific manner. Overall, we found that female mice exhibit unique patterns of differentially expressed genes throughout AP infection compared to male mice and that nociceptors regulate the expression of these genes. Additionally, nociceptor ablation results in a greater enrichment of biological processes related to immune responses earlier compared to cre-control females and greater expression of genes involved in inflammatory processes and osteolytic activity. Therefore, while nociceptor ablation augments inflammatory and bone resorption responses in both males and females in a mouse model of AP, transcriptomic analyses demonstrate that the mechanisms through which nociceptors modulate AP are distinct between sexes.

Initial comparisons between nociceptor-ablated and cre-control periapical lesion transcriptomes revealed that nociceptor-ablated females expressed a greater number of differentially expressed genes after 14 days of infection. Notably, nociceptors regulated the expression of key genes in Wnt signaling pathways, a mechanism known to be crucial in bone development and homeostasis (González-Quintanilla et al., 2021). After 14 days of infection, nociceptor-ablated females exhibited greater levels of *Wnt7b* and *Wnt4*, whereas cre-control females expressed more significant levels of *Dkk1*. There is growing evidence that both *Wnt7b* and *Wnt4* promote the differentiation of stem cells within dental tissues, and *Wnt4* has also been shown to inhibit osteoclast formation (Lv et al., 2018; Chen et al., 2019; Xu et al., 2022). Conversely, *Dkk1* is a secreted inhibitor of Wnt signaling and known to play a role in negative feedback within this pathway (Cao et al., 2015). These findings suggest that at day 14, there is activation of bone mineralization differentiation via Wnt signaling, and nociceptor ablation could inhibit early stimulation of these pathways since cre-control females are already showing evidence of the negative feedback of Wnt signaling. Interestingly, both nociceptor-ablated and cre-control mice exhibited upregulation of *Sfrp4*, another Wnt inhibitor, from days 0 to 7 and 0 to 14 (Haraguchi et al., 2016). However, nociceptor-ablated females exhibited a greater fold change in this gene, suggesting this could account for early inhibition of Wnt signaling pathway activation in nociceptor-ablated mice. As such, we have demonstrated the regulation of Wnt-related gene transcription by nociceptors in female mice modeling AP.

While Wnt signaling is a key pathway in bone formation, there are a variety of other genes regulated by nociceptors related to bone homeostasis in AP. When examining changes in gene expression from day 0 to 14, both nociceptor-ablated and cre-control mice showed upregulation of *Tnfsf11*, the gene encoding RANKL, the cytokine necessary for osteoclast differentiation (Benedetto et al., 2013). However, the fold change in *Tnfsf11* expression was greater in nociceptor-ablated mice, reinforcing increases in osteolytic activity we have previously shown in nociceptor-ablated mice (Austah et al., 2022). Other genes known to promote bone resorption, including *Tnfrsf11a* (encodes RANK), *Mmp9*, *Ctsk*, and *Acp5* (encodes TRAP), were exclusively upregulated in cre-control mice between 0 and 7 days, suggesting an earlier osteolytic response in cre-control mice. Conversely, over the course of 14 days, there was significant downregulation of osteoblast genes in both strains, including *Dspp* and *Fgf9*, with their fold changes being greater in cre-control mice. Moreover, nociceptor-ablated mice exhibited exclusive downregulation of *Sost*, while *Bmp7* was only downregulated in cre-control females. Furthermore, we have shown that cre-control females demonstrated a tremendous increase in *Prl* and *Gh* (both ligands of the prolactin receptor) expression throughout AP progression, whereas nociceptor-ablated females showed much lower expression of these two genes. Male mice exhibited minimal *Prl* and *Gh* transcription in periapical lesions. This is particularly of interest, given the growing understanding of the role of prolactin receptor signaling in bone homeostasis, inflammation, and pain in a sex-specific manner (Ledesma-Colunga et al., 2017; Chen et al., 2020; Paige et al., 2020). Together, these results suggest that nociceptors regulate the kinetics of bone homeostasis genes, inhibiting mineralization and promoting bone resorption, throughout AP infection.

Furthermore, we have demonstrated that nociceptors modulate the kinetics of gene expression related to immune and inflammatory processes. Over 14 days of infection, nociceptor-ablated females consistently showed a greater fold enrichment of biological processes, including those related to inflammatory response and immune cell differentiation, activation, and chemotaxis. However, we have shown that while these processes had a greater enrichment in nociceptor-ablated mice early on the infection (i.e., day 0–7), cre-control mice showed a greater enrichment of these processes later in AP progression (i.e., day 7–14). This complements our early work demonstrating nociceptor ablation results in an earlier influx of immune cells in AP (Austah et al., 2022). Notably, at day 14, both nociceptor-ablated females and males exhibited greater expression of chemokines, including *Cxcl1*, *Cxcl2*, and *Cxcl3*, suggesting that increased chemotaxis is a common consequence of nociceptor ablation between males and females. Key AP inflammatory genes, including *Tnf* and *Il1b*, are also tightly regulated by nociceptors in a sex-specific manner. Nociceptor-ablated males exhibited greater expression of these two genes at 14 days compared to cre-control males. In contrast, nociceptor-ablated females demonstrated exclusive upregulation of these genes between days 0 and 7 compared to cre-control females. These findings demonstrate that nociceptor ablation results in an enhanced inflammatory response in both males and females, but the targets and kinetics of modulation differ between sexes.

In summary, we have shown that males and females demonstrate unique transcriptomic profiles within the periapical lesions of AP infections. Furthermore, nociceptors sex specifically modulate gene expression, particularly related to osteolytic activity and inflammatory processes. These studies will further provide the foundation needed to study mechanisms of sex differences in AP. Understanding these mechanisms can ultimately inform treatment options to alleviate suffering for millions of patients suffering from AP.

## Data Availability

The datasets generated during and/or analyzed during the current study are available in the GEO repository (GSE254607) https://www.ncbi.nlm.nih.gov/geo/query/acc.cgi?acc=GSE254607.

## References

[B1] Austah O. N. Lillis K. V. Akopian A. N. Harris S. E. Grinceviciute R. Diogenes A. (2022). Trigeminal neurons control immune-bone cell interaction and metabolism in apical periodontitis. Cell. Mol. Life Sci. 79 (6), 330. 10.1007/s00018-022-04335-w 35639178 PMC9156470

[B2] Benedetto A. D. Gigante I. Colucci S. Grano M. (2013). Periodontal disease: linking the primary inflammation to bone loss. Clin. Dev. Immunol. 2013, 1–7. 10.1155/2013/503754 PMC3676984 23762091

[B3] Berlinck T. Tinoco J. Carvalho F. Sassone L. Tinoco E. (2015). Epidemiological evaluation of apical periodontitis prevalence in an urban brazilian population. Braz. Oral Res. 29, 51. 10.1590/1807-3107BOR-2015.vol29.0051 25760068

[B4] Bonnot T. Gillard M. B. Nagel D. H. (2019). A simple protocol for informative visualization of enriched gene ontology terms. BIO-PROTOCOL 9. 10.21769/bioprotoc.3429

[B5] Cao Z. Liu R. Zhang H. Liao H. Zhang Y. Hinton R. J. (2015). Osterix controls cementoblast differentiation through downregulation of wnt-signaling via enhancing dkk1 expression. Int. J. Biol. Sci. 11 (3), 335–344. 10.7150/ijbs.10874 25678852 PMC4323373

[B6] Chen D. Yu F. Wu F. Bai M. Lou F. Liao X. (2019). The role of wnt7b in the mediation of dentinogenesis via the erk1/2 pathway. Archives Oral Biol. 104, 123–132. 10.1016/j.archoralbio.2019.05.009 31181411

[B7] Chen Y. Navratilova E. Dodick D. W. Porreca F. (2020). An emerging role for prolactin in female-selective pain. Trends Neurosci. 43 (8), 635–648. 10.1016/j.tins.2020.06.003 32620290

[B8] Estrela C. Guedes O. A. Silva J. A. Leles C. R. Estrela C. R. Pecora J. D. (2011). Diagnostic and clinical factors associated with pulpal and periapical pain. Braz Dent. J. 22 (4), 306–311. 10.1590/s0103-64402011000400008 21861030

[B9] Garlet G. P. (2010). Destructive and protective roles of cytokines in periodontitis: a re-appraisal from host defense and tissue destruction viewpoints. J. Dent. Res. 89 (12), 1349–1363. 10.1177/0022034510376402 20739705

[B10] González-Quintanilla D. Abásolo N. Astudillo P. (2021). Wnt signaling in periodontal disease. Front. Dent. Med. 2. 10.3389/fdmed.2021.763308

[B11] Graunaite I. Lodiene G. Maciulskiene V. (2012). Pathogenesis of apical periodontitis: a literature review. J. Oral Maxillofac. Res. 2 (4), e1. 10.5037/jomr.2011.2401 24421998 PMC3886078

[B12] Haraguchi R. Kitazawa R. Mori K. Tachibana R. Kiyonari H. Imai Y. (2016). Sfrp4-dependent wnt signal modulation is critical for bone remodeling during postnatal development and age-related bone loss. Sci. Rep. 6 (1), 25198. 10.1038/srep25198 27117872 PMC4846872

[B13] Khan A. A. Owatz C. B. Schindler W. G. Schwartz S. A. Keiser K. Hargreaves K. M. (2007). Measurement of mechanical allodynia and local anesthetic efficacy in patients with irreversible pulpitis and acute periradicular periodontitis. J. Endod. 33 (7), 796–799. 10.1016/j.joen.2007.01.021 17804314

[B14] Ledesma-Colunga M. G. Adan N. Ortiz G. Solis-Gutierrez M. Lopez-Barrera F. Martinez de la Escalera G. (2017). Prolactin blocks the expression of receptor activator of nuclear factor κB ligand and reduces osteoclastogenesis and bone loss in murine inflammatory arthritis. Arthritis Res. Ther. 19 (1), 93. 10.1186/s13075-017-1290-4 28506283 PMC5433139

[B15] Lillis K. V. Austah O. Grinceviciute R. Garlet G. P. Diogenes A. (2023). Nociceptors regulate osteoimmune transcriptomic response to infection. Sci. Rep. 13 (1), 17601. 10.1038/s41598-023-44648-9 37845223 PMC10579402

[B16] Lv H. Yang J. Wang C. Yu F. Huang D. Ye L. (2018). The wnt7b protein promotes the migration and differentiation of human dental pulp cells partly through wnt/beta-catenin and c-jun n-terminal kinase signalling pathways. Archives Oral Biol. 87, 54–61. 10.1016/j.archoralbio.2017.12.015 29268145

[B17] Mecklenburg J. Zou Y. Wangzhou A. Garcia D. Lai Z. Tumanov A. V. (2020). Transcriptomic sex differences in sensory neuronal populations of mice. Sci. Rep. 10 (1), 15278. 10.1038/s41598-020-72285-z 32943709 PMC7499251

[B18] Mi H. Muruganujan A. Huang X. Ebert D. Mills C. Guo X. (2019). Protocol update for large-scale genome and gene function analysis with the panther classification system (v.14.0). Nat. Protoc. 14 (3), 703–721. 10.1038/s41596-019-0128-8 30804569 PMC6519457

[B19] Mohaved S. B. Shilpa G. Li Q. Austah O. Bendele M. Brock R. (2020). Apical periodontitis-induced mechanical allodynia: a mouse model to study infection-induced chronic pain conditions. Mol. Pain 16, 1744806919900725. 10.1177/1744806919900725 31902318 PMC6977224

[B20] Nusstein J. M. Beck M. (2003). Comparison of preoperative pain and medication use in emergency patients presenting with irreversible pulpitis or teeth with necrotic pulps. Oral Surg. Oral Med. Oral Pathol. Oral Radiol. Endod. 96 (2), 207–214. 10.1016/s1079-2104(02)91732-4 12931095

[B21] Paige C. Barba-Escobedo P. A. Mecklenburg J. Patil M. Goffin V. Grattan D. R. (2020). Neuroendocrine mechanisms governing sex differences in hyperalgesic priming involve prolactin receptor sensory neuron signaling. J. Neurosci. 40 (37), 7080–7090. 10.1523/JNEUROSCI.1499-20.2020 32801151 PMC7480243

[B22] Polycarpou N. Ng Y. L. Canavan D. Moles D. R. Gulabivala K. (2005). Prevalence of persistent pain after endodontic treatment and factors affecting its occurrence in cases with complete radiographic healing. Int. Endod. J. 38 (3), 169–178. 10.1111/j.1365-2591.2004.00923.x 15743420

[B23] Schmittgen T. D. Livak K. J. (2008). Analyzing real-time pcr data by the comparative c(t) method. Nat. Protoc. 3 (6), 1101–1108. 10.1038/nprot.2008.73 18546601

[B24] Silva T. A. Garlet G. P. Fukada S. Y. Silva J. S. Cunha F. Q. (2007). Chemokines in oral inflammatory diseases: apical periodontitis and periodontal disease. J. Dent. Res. 86 (4), 306–319. 10.1177/154405910708600403 17384024

[B25] Stirling L. C. Forlani G. Baker M. D. Wood J. N. Matthews E. A. Dickenson A. H. (2005). Nociceptor-specific gene deletion using heterozygous nav1.8-cre recombinase mice. Pain 113 (1-2), 27–36. 10.1016/j.pain.2004.08.015 15621361

[B26] Xu W. Lu Q. Qu M. Fan R. Leng S. Wang L. (2022). Wnt4 regulates bone metabolism through ikk-nf-κb and rock signaling under occlusal traumatic periodontitis. J. Periodontal Res. 57 (3), 461–469. 10.1111/jre.12975 35137408

[B27] Yucel-Lindberg T. Båge T. (2013). Inflammatory mediators in the pathogenesis of periodontitis. Expert Rev. Mol. Med. 15, e7. 10.1017/erm.2013.8 23915822

